# Role of intravenous alteplase on late lesion growth and clinical outcome after stroke treatment

**DOI:** 10.1177/0271678X231167755

**Published:** 2023-04-05

**Authors:** Praneeta Konduri, Fabiano Cavalcante, Henk van Voorst, Leon Rinkel, Manon Kappelhof, Katinka van Kranendonk, Kilian Treurniet, Bart Emmer, Jonathan Coutinho, Lennard Wolff, Jeanette Hofmeijer, Maarten Uyttenboogaart, Wim van Zwam, Yvo Roos, Charles Majoie, Henk Marquering, on behalf of the MR CLEAN-NO IV Trial Investigators (Multicenter Randomized Clinical Trial of Endovascular Treatment for Acute Ischemic Stroke in the Netherlands)

**Affiliations:** 1 Department of Biomedical Engineering and Physics, Amsterdam UMC, location University of Amsterdam, Amsterdam, the Netherlands; 2 Department of Radiology and Nuclear Medicine, Amsterdam UMC, location University of Amsterdam, Amsterdam, the Netherlands; 3 Department of Neurology, Amsterdam UMC, location University of Amsterdam, Amsterdam, the Netherlands; 4 Department of Radiology, Haaglanden MC, The Hague, The Netherlands; 5 Department of Radiology & Nuclear Medicine, Erasmus MC, University Medical Center, Rotterdam, Netherlands; 6 Department of Neurology, Rijnstate Hospital, Arnhem, the Netherlands; 7 Department of Clinical Neurophysiology, University of Twente, Enschede, the Netherlands; 8 Department of Neurology, University Medical Center Groningen, Groningen, the Netherlands; 9 Department of Radiology and Nuclear Medicine, Maastricht University Medical Center and Cardiovascular Research Institute Maastricht (CARIM), Maastricht, the Netherlands

**Keywords:** Brain edema, intracranial/intracerebral hemorrhage, neurovascular coupling, TPA, acute stroke

## Abstract

Several acute ischemic stroke mechanisms that cause lesion growth continue after treatment which is detrimental to long-term clinical outcome. The potential role of intravenous alteplase treatment (IVT), a standard in stroke care, in cessing the physiological processes causing post-treatment lesion development is understudied. We analyzed patients from the MR CLEAN-NO IV trial with good quality 24-hour and 1-week follow-up Non-Contrast CT scans. We delineated hypo- and hyper-dense regions on the scans as lesion. We performed univariable logistic and linear regression to estimate the influence of IVT on the presence (growth > 0 ml) and extent of late lesion growth. The association between late lesion growth and mRS was assessed using ordinal logistic regression. Interaction analysis was performed to evaluate the influence of IVT on this association. Of the 63/116 were randomized to included patients, IVT. Median growth was 8.4(−0.88–26) ml. IVT was not significantly associated with the presence (OR: 1.24 (0.57–2.74, p = 0.59) or extent (β = 5.1(−8.8–19), p = 0.47) of growth. Late lesion growth was associated with worse clinical outcome (aOR: 0.85(0.76–0.95), p < 0.01; per 10 ml). IVT did not influence this association (p = 0.18). We did not find evidence that IVT influences late lesion growth or the relationship between growth and worse clinical outcome. Therapies to reduce lesion development are necessary.

## Introduction

Ischemic lesions do not stabilize even after treatment of acute ischemic stroke (AIS) either with intravenous alteplase treatment (IVT) or with endovascular treatment (EVT). It has been shown that ischemic lesions may continue to evolve at least between 24 hours and 1 week after the onset of AIS.^1,2^ In a subpopulation of the MR CLEAN trial that received follow-up Non-Contrast CT (NCCT) images 24 hours and 1 week after stroke, it was found that late lesion growth consisting of edema, infarct and hemorrhagic growth was associated with an unfavorable functional outcome. Moreover, successfully restoring blood flow did not influence the association between late lesion growth and functional outcome.^1^ Various physiological processes may play a role in lesion growth even after treatment including excitotoxicity, oxidative stress, breakdown of the blood-brain barrier, microvascular damage, and inflammation.^1,3^

The current standard of care for treating AIS due to a large vessel occlusion consists of the IVT followed by endovascular treatment (EVT).^4^ The clinical benefits of treatment with IVT, especially when it can promptly and successfully lyse the clot have been established. It can help to dissolve residual and distal thrombi, and aid in salvaging the ischemic tissue. However, previous studies have suggested that tissue plasminogen activator (tPA) may also be neurotoxic and render the tissue more susceptible to edema and hemorrhage.^4,5^ The influence of IVT on the subacute lesion growth in humans is not fully understood. Identifying the relationship between IVT and lesion growth, can help to understand if IVT augments the post-treatment patho-physiological cascade and better target neuroprotective and secondary treatments.

The aim of this study was to evaluate the influence of IVT on late lesion growth between 24 hours and 1 week after onset of an AIS due to a large vessel occlusion in the anterior circulation in patients treated with EVT. Furthermore, we aimed to validate the previously shown association between late lesion growth and outcome after 90 days on our study population and assess influence of IVT on this association.

## Materials and methods

### Patient population

In this sub-study, we used data of patients included in the MR CLEAN-NO IV trial. This was an open-label, multicenter, randomized trial in Europe that assessed the value of IVT before endovascular treatment (EVT) of patients with acute ischemic stroke due to a proximal intracranial large vessel occlusion. Patients above the age of 18 years, who were eligible for IVT with alteplase within 4.5 hours of symptom onset and presented directly at a center providing both treatments were randomized to receive either EVT alone or EVT after IVT. Information regarding the inclusion and exclusion criterion employed by the trial have been published previously.^5^ The trial protocol mandated either a Non-Contrast Computed Tomography (NCCT) after 24 hours and 1 week or a Magnetic Resonance Imaging (MRI) after 24 hours, based on center-specific capabilities/patient contraindications.^6^ In this study, we assessed patients that received a NCCT scan after 24 hours and 1 week after EVT. We excluded patients with clear evidence of extensive contrast extravasation, poor quality scans that were incomplete or included movement artefacts, beam-hardening effects, and other technical errors. We also excluded patients that required decompressive craniectomy to only evaluate lesion growth in patients without a malignant swelling. Details of the inclusion and exclusion criterion used in this study are provided in the Supplementary Figure 1. A STROBE checklist is also provided in the Supplementary Material.

The MR CLEAN-NO IV protocol was approved in the Netherlands by the central medical ethics committee and research board of the Erasmus MC University Medical Center, Rotterdam, the Netherlands (MEC-2017-368) before start of the trial. In France, the study was approved by the Comité de Protection des Personnes, Ile de France IV (ID-RCB: 2018-A00764-51). In Belgium, the study was approved by the Central Ethics Committee Research UZ/KU Leuven, Belgian Registration Number: B322201939935, as well as the Comité d’Ethique Medicale, CHC, Liège, Belgium (study number: 19/20/987). The study was conducted according to the principles of the Declaration of Helsinki (7th revision, October 2013), ICH-GCP, the Dutch Medical Research Involving Human Subjects Act (WMO) and when applicable in accordance with regulations of other countries with participating centers. The trial protocol, including protocol version and amendments, can be found on the website https://www.mrclean-noiv.nl. The trial used a deferred consent procedure in accordance with national legislation in the participating countries. Patients or their legal representatives provided written informed consent. Patient data can be provided on request to MR CLEAN-NO IV trial committee.^5^

### Image analysis

Ischemic lesions and hemorrhagic areas were segmented using a deep-learning based software developed by Nico.lab.^7^ The segmented lesions were manually corrected by trained observers (F. Calvacante and P. Konduri) using a fixed window width of 40 Hounsfield units and center-level setting of 40 Hounsfield units on ITK-Snap Software.^8^ The trained observers were blinded to all clinical data other than occlusion location during the delineation process. The ischemic lesions included edema and brain swelling extending into the contralateral hemisphere or resulting in sulcal or ventricular effacement and hyper densities suspected as hemorrhagic transformation or contrast extravasation within or around the identified hypo-dense areas. Chronic lesions showing fluid attenuation, distinct borders and/or volume loss were excluded from the delineation. The hemorrhagic region was only identified in patients that presented hemorrhagic transformation on the 1-week follow-up NCCT scans as determined by the imaging core lab. The segmentations were verified by comparing their locations with the ASPECTS scores graded by the imaging core lab of the MR CLEAN-NO IV trial and an experienced neuro-radiologist (C. Majoie).

Lesion volumes were obtained by determining the product of the number of voxels within the delineation and the voxel size. Non-hemorrhagic (infarct and edema) volume was defined as the volume of the hypo-dense areas within the lesion, excluding the volume of the hemorrhagic regions. In total we assessed three lesion volumes: non-hemorrhagic, hemorrhagic, and combined volumes. Growth was defined as the difference between these volumes at 1 week and 24 hours resulting in late lesion, non-hemorrhagic and hemorrhagic growth, respectively.

### Statistics analysis

We first compared the differences in late lesion, non-hemorrhagic and hemorrhagic growth between the patients that were randomized to receive IVT with EVT or direct EVT using Mann-Whitney U test. Next, we determined the association between (intention to) administering IVT and lesion, non-hemorrhagic or hemorrhagic evolution using univariable linear regression. We investigated the influence of successful treatment (eTICI: 2 b-3) on the lesion, non-hemorrhagic and hemorrhagic volumes on the complete study population and within each of the treatment arms.

Primary outcome was the 90-day modified Rankin Scale (mRS) and the secondary outcome was defined as favorable functional outcome (mRS ≤ 2). We assessed the influence of late lesion, non-hemorrhagic, and hemorrhagic growth on functional outcome using univariable and multivariable ordinal logistic regression after adjusting for confounders (baseline, clinical and imaging characteristics that were associated with functional outcome at the significance level of p < 0.1). To assess the influence of IVT on these associations, we performed unadjusted interaction analysis by introducing a multiplicative term of IVT with the parameter under consideration. We also repeated these analyses with favorable functional outcome as the outcome variable. Lastly, to remove the confounding effect of hemorrhagic transformation on the association between late lesion growth and outcome, we performed univariable and multivariable ordinal regression in the sub-group of patients that did not suffer from a hemorrhagic transformation.

Statistical analyses were performed using SPSS (IBM SPSS Statistics, version 26, 2019) and R (Version 4.0.2 [2020-06-22]) using RStudio (Version 1.2.5033 2009–2019 RStudio, Inc.) packages: MASS, dplyr, reshape, ggeffects, foreign, ggplot2, DescTools, ggpubr. A P ≤ 0.05 was considered statistically significant.

## Results

### Patient population

Of the 133 patients that received both 24-hour and 1-week NCCT scans in the MR CLEAN-NO IV trial, we excluded patients with poor image quality (n = 10), old lesions (n = 5) and other complications (sub-arachnoid hemorrhage: n = 1 and craniectomy: n = 1); thus, analyzing 116 patients in this study. (Flowchart is presented in the Supplementary Material.) The median age of the included population was 71 (IQR: 59–76) years, and 43 (37%) patients were female. Eleven percent of the population had suffered from a previous stroke and 18% presented a history of diabetes mellitus. The median NIH Stroke Scale (NIHSS) and ASPECTS at baseline were 16 (IQR: 11–19) and 9 (IQR: 8–10), respectively. Fifty-three (46%) patients were randomized to undergo direct EVT without the administration of intravenous (IVT) thrombolytic. Successful reperfusion (eTICI:2b-3) was achieved in 90 (87%) patients and 57 (49%) patients achieved a favorable functional outcome. Baseline characteristics of the patients included in this study are provided in Table 1.

**Table 1.** Baseline, imaging, treatment, and post-treatment characteristics along with a comparison between patients randomized to receive or not receive iv-TPA before IAT.

Characteristics	Population N = 116	Treatment allocation		p-value
		IVT and IAT	Direct IAT	
Age	71 (59–76)	66 (58–75)	73 (63–79)	0.08
Male sex	73 (63%)	39 (62%)	34 (64%)	0.95
*Clinical characteristics*				
Previous ischemic stroke	13 (11%)	7 (11%)	6 (11)	1.00
Atrial fibrillation	18 (16%)	9 (14%)	9 (17%)	0.89
Diabetes mellitus	21 (18%)	10 (16%)	11 (21%)	0.66
Hypertension	50 (43%)	27 (43%)	23 (43%)	1.00
Pre-treatment mRS >2				
Summary	3 (2.6%)	2 (3.2%)	1 (2%)	1.00
Missing	1 (0.86%)			
Glucose				
Summary	6.6 (5.9–8.1)	6.6 (5.9–8.7)	6.5 (6–7.8)	0.60
Missing	3 (2.6%)			
Systolic blood pressure (mmHg)	150 (130–170)	150 (140–170)	150 (120–170)	0.31
Baseline NIH Stroke Scale	16 (11–19)	16 (9–19)	16 (11–19)	0.73
*Imaging characteristics*				
ASPECTS	9 (8–10)	9 (8–10)	9 (8–10)	0.84
Collateral score				
Absent	6 (5.3%)	3 (4.9%)	3 (5.7%)	0.95
Filling <50% of occluded area	31 (27%)	17 (28%)	14 (26%)	
Filling >50% and <100% of occluded area	51 (45%)	26 (43%)	25 (47%)	
Filling 100% of occluded area	26 (23%)	15 (25%)	11 (21%)	
Missing	2 (1.7%)			
Right hemisphere stroke	57 (49%)	29 (46%)	28 (53%)	0.59
Non-proximal occlusion	96 (83%)	54 (86%)	42 (79%)	0.50
*Treatment characteristics*				
Onset to randomization	92 (70–140)	88 (70–150)	98 (70–140)	0.93
Onset to needle (minutes)				
Summary	92 (78–140)	92 (77–140)	120 (110–120)	0.57
Missing	55 (47%)			
Door to groin (minutes)				
Summary	68 (53–90)	64 (55–86)	70 (53–94)	0.65
Missing	5 (4.3%)			
Door to needle (minutes)				
Summary	32 (24–40)	32 (24–40)	30 (18–42)	0.87
Missing	55 (47%)			
Onset to groin (minutes)				
Summary	140 (110–190)	140 (110–220)	150 (110–190)	0.87
Missing	5 (4.3%)			
Onset to reperfusion (minutes)				
Summary	180 (150–230)	180 (150–220)	180 (150–240)	0.94
Missing	29 (25%)			
*Post-treatment characteristics*				
Successful recanalization				
Summary	90 (87%)	47 (84%)	43 (90%)	0.58
Missing	12 (10%)			
Favourable functional outcome	57 (49%)	33 (52%)	24 (45%)	0.57
modified Rankin Scale				
0	3 (26%);	2 (3.2%);	1 (1.9%);	0.46
1	10 (9%);	4 (6.3%);	6 (11%);	
2	44 (38%);	27 (43%);	17 (32%);	
3	11 (9.5%)	4 (6.3%);	7 (13%)	
4	18 (16%)	12 (19%)	6 (11%)	
5	15 (13%)	8 (13%)	7 (13%)	
6	15 (13%)	6 (9.5%)	9 (17%)	

Data are displayed as median (interquartile range) or number (%population). Missing information, when applicable, is also provided as number (%population). Mann-Whitney U test and Chi-Square/Fisher tests were performed to compare continuous and binary/categorical variables between the sub-groups based on treatment allocation appropriately.

The baseline, treatment, and follow-up characteristics of the patients included in this study are comparable to those of the complete MR CLEAN-NO IV trial population. (Supplementary Table 1). Furthermore, these characteristics were also comparable between patients included in this study and those that received the MRI-workflow at follow-up. (Supplementary Table 2).

### Lesion characteristics

**Figure 1.**
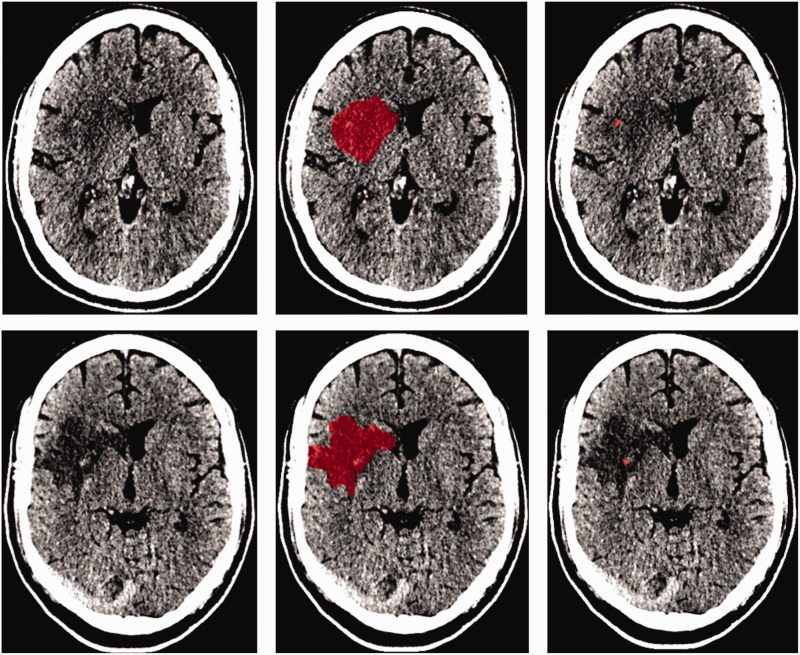
Example of a 24-hour (top) and 1-week (bottom) image of a patient with a hemorrhagic transformation identified on the 1-week scan. The center columns represent the lesion segmentations in red and the right column represents the hyperdensities suspected of hemorrhage.

**Figure 2.**
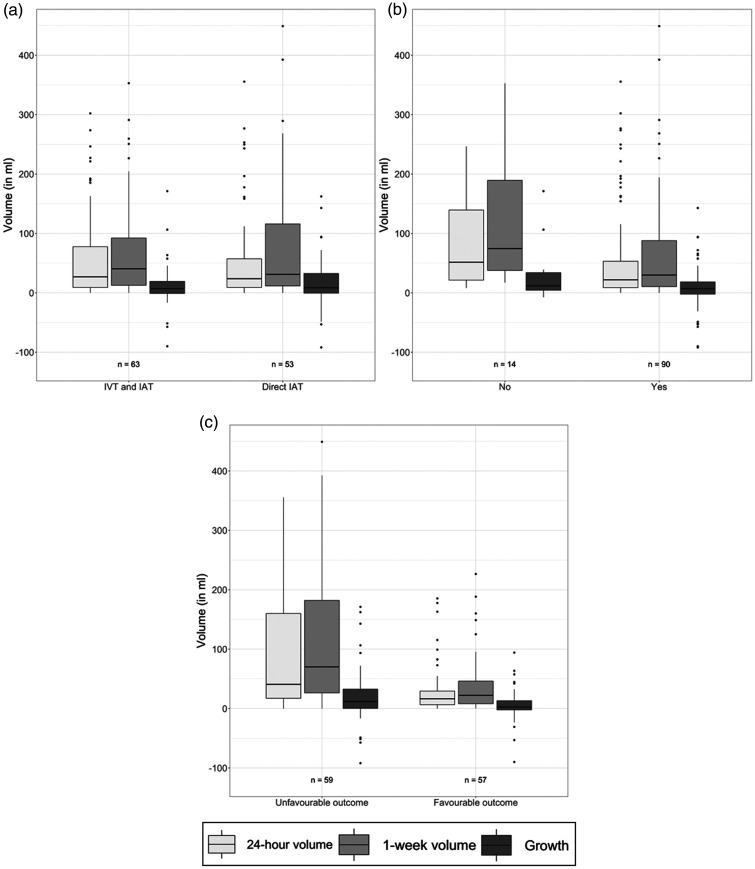
Boxplots comparing the 24-hour and 1 week lesion volumes and late lesion growth (24-hours to 1 week after stroke onset) between subgroups based on, (a) treatment allocation, (b) successful treatment (eTICI ≥ 2b) and, (c) favourable functional outcome (mrs ≤ 2).

An example of the lesion segmentation on the 24-hour and 1-week scan is provided in Figure 1. In our population, the median lesion volumes after 24 hours and 1 week were 25 (IQR: 9.1–70) ml and 37 (IQR: 12–110) ml, respectively. Forty-one (35%) patients had hemorrhagic transformation with a median hemorrhage volume of 1.7 (IQR: 0.13–5.46) ml and 0.93 (IQR: 0.3–2.9) ml after 24 hours and 1 week, respectively. Wilcoxon signed ranked test showed that the lesion and non-hemorrhagic volumes were significantly larger at one week compared to 24 hours after stroke. Seventy-eight (67%) patients had late lesion growth and the median late lesion growth was 8.4 (IQR: −0.88–26) ml. Details of the lesion characteristics are provided in Figure 2 and the Supplementary Table 3.

### IVT with alteplase and lesion evolution

The median lesion and non-hemorrhagic volumes after 24 hours in the patients that received IVT (27 (IQR: 9.2–78) ml and 24 (IQR: 9–77) ml, respectively) and in those that underwent direct EVT (24 (IQR: 9.1–57) ml and 24 (IQR: 9.1–53) ml, respectively) were not significantly different (p = 0.87, p = 0.94, respectively). Similarly, the lesion and non-hemorrhagic volumes after 1 week were similar between patients with and without IVT (41(IQR: 13–92) ml vs. 31 (IQR: 12–120); p = 0.71 and 40 (IQR: 13–90) ml vs. 31 (IQR: 12–120) ml; p = 0.72, respectively). Furthermore, there were no significant differences in the late lesion and non-hemorrhagic growth between patients with and without IVT (7.1 (IQR: −0.94–19) ml vs. 8.7 (IQR: −0.53–33) ml p = 0.59 and 9.2 (IQR: −0.41–23) ml vs. 8.7 (IQR: −0.53−32) ml p = 0.84, respectively). Details of the lesion characteristics are provided in Figure 2.

Univariable linear regression showed no significant association between IVT and late lesion growth (β = 5.1 (95% CI: -8.8–19), p = 0.47) and late non-hemorrhagic growth (β = 4.0 (95% CI:-9.7–18), p = 0.56). Furthermore, IVT was not statistically significantly associated with presence of late lesion growth >0 ml (OR: 1.24 (95% CI: 0.57–2.74, p = 0.59) and late non-hemorrhagic growth >0 ml (OR: 1.00 (95% CI:0.45–2.23, p = 1.00).

### Treatment outcome and lesion evolution

In our population, ninety (87%) of the patients achieved successful recanalization (eTICI ≥ 2b). Lesion and non-hemorrhagic volumes after 24 hours and 1 week were significantly lower in patients that achieved successful recanalization. Lesion and non-hemorrhagic growth were comparable in patients with and without successful recanalization. Details of these comparisons are provided in Figure 2 and Supplementary Table 3.

The treatment success rates were similar in patients receiving IVT before EVT and patients that underwent direct EVT (47(84%) vs. 43 (90%), p = 0.58). All lesion, non-hemorrhagic and hemorrhagic characteristics were comparable between patients that did and do not achieve successful recanalization in the direct EVT treatment arm. However, the 1-week lesion and non-hemorrhagic volume were significantly larger in patients that did not achieve reperfusion in the IVT before EVT treatment arm (p = 0.04, p = 0.03). Details of these comparisons are provided in the Supplementary Table 3.

### Lesion evolution and functional outcome

Lesion and non-hemorrhagic volumes after 24 hours and 1 week were significantly lower in patients that achieved favorable functional outcome compared to those that did not (16 (6.5–29), 41 (17–160) ml, p < 0.01; 22 (8.1–46), 70 (26–180) ml, p < 0.01 and 16 (5.9–29) ml, 41 (17–150) ml, p < 0.01) and 1 week (22 (8.1–46) ml, 66 (26–180) ml, p < 0.01, respectively). Furthermore, patients that achieved favorable functional outcome presented significantly lower late lesion growth (2.5 (IQR: −2.1–13) ml, 12 (IQR: 0.36–33) ml, p = 0.02) and non-hemorrhagic growth (2.5 (IQR: −1.9–13) ml, 13 (IQR: 2.1–33) ml, p < 0.01). Details of the lesion characteristics are provided in Figure 2 and the non-hemorrhagic and hemorrhagic characteristics are provided in the Supplementary Figure 2 and Supplementary Figure 3, respectively.

Late lesion and non-hemorrhagic growth were associated with a worse functional outcome before and after adjusting for potential confounders (OR: 0.87 (95% CI: 0.79–0.96), p = 0.01, aOR: 0.85 (95% CI: 0.76–0.95), p < 0.01; per 10 ml) and (OR: 0.86 (95% CI: 0.78–0.95), p < 0.01, aOR: 0.84 (95% CI: 0.75–0.94), p < 0.01; per 10 ml). This trend was not observed in the association of hemorrhagic growth and functional outcome (OR: 1.11 (95% CI: 0.98–1.25) p = 0.12, aOR: 1.11 (95% CI: 0.90–1.36), p = 0.34 per ml). In the subgroup of patients that did not suffer from hemorrhagic transformation, subacute lesion growth was associated with worse functional outcome, in both univariable (0.76 (95% CI: 0.62–0.90), p < 0.01) and multivariable ordinal regression analysis (aOR: 0.66 (95% CI: 0.51–0.81), p < 0.01). Details of the multivariable ordinal regression models are provided in Table 2. Details of the subgroup analysis on patients that did not suffer from hemorrhagic transformation and those on the confounder selection are provided in the Supplementary Table 4 and Supplementary Table 5, respectively.

**Table 2.** Multivariable ordinal logistic regression models showing the association of late lesion and non-hemorrhagic growth between 24 hours and 1 week after EVT and modified Rankin Score after 90 days.

Variable	Model 1: Late lesion growth		Model 2: Late non-hemorrhagic growth	
	Odds-Ratio (95% CI)	p-value	Odds-Ratio (95% CI)	p-value
Growth^	0.85 (0.76–0.95)	<0.01^**^	0.84 (0.75–0.94)	<0.01**
24-hour lesion volume^	0.86 (0.81–0.91)	<0.01^**^	0.86 (0.81–0.91)	<0.01**
Age	0.95 (0.92–0.98)	<0.01**	0.95 (0.92–0.98)	<0.01**
Sex (Male)	2.3 (0.98–5.3)	0.06⨚	2.3 (0.99–5.4)	0.05*
Previous ischemic stroke	0.38 (0.10–1.41)	0.15	0.37 (0.10–1.39)	0.14
Atrial fibrillation	0.77 (0.25–2.44)	0.66	0.78 (0.25–2.45)	0.66
Systolic blood pressure (mmHg)	1.00 (0.98–1.01)	0.61	1.0 (0.98–1.01)	0.64
Onset to randomization (minutes)	0.99 (0.98–1.00)	0.02*	0.99 (0.98–1.00)	0.02*
Successful reperfusion	2.39 (0.78–7.39)	0.13	2.38 (0.78–7.36)	0.13

^^^Analysis done for 10 ml volume; **p ≤ 0.01, *p ≤ 0.05, ⨚p ≤ 0.10; Patients with missing information for a variable were excluded from the analysis.

Interaction analysis, by introducing a multiplicative term, showed no influence of IVT on the association of late lesion (p = 0.18), non-hemorrhagic (p = 0.28) or hemorrhagic (p = 0.93) growth on functional outcome. Details of the interaction analysis are provided in the Supplementary Table 6.

Lastly, late lesion and non-hemorrhagic growth were associated with unfavorable functional outcome (mRS ≤ 2) in univariable binary regression (OR: 0.89 (95% CI: 0.78–0.99), p = 0.05; OR: 0.88 (95% CI: 0.77–0.98), p = 0.03, respectively; per 10 ml) but lost significance after adjusting for confounders (aOR: 0.84 (95% CI: 0.69–1.00), p = 0.07; OR: 0.83 (95% CI: 0.68–0.99), p = 0.06, respectively; per 10 ml). Hemorrhagic growth was not associated with favorable functional outcome (OR: 1.10 (95% CI: 0.91–1.32), p = 0.32). Furthermore, there was no significant influence of IVT on the association between late lesion, non-hemorrhagic, or hemorrhagic growth on favorable functional outcome (OR (0.94 (95% CI: 0.74–1.19) per 10 ml, p = 0.60; OR: 0.97 (95% CI: 0.76–1.25) per 10 ml, p = 0.80; OR: 0.86 (95% CI: 0.47–1.56) per ml, p = 0.61; respectively). Details of the binary logistic regression analysis are provided in the Supplementary Table 6 and Supplementary Table 7).

## Discussion

In this study where patients were randomized to receive or not receive IVT before EVT, we showed no significant association between treatment with IVT before EVT with the occurrence or extent of late lesion growth in the post-treatment subacute period (between 24 hours and 1 week). The lesion continues to evolve after treatment, which is line with previous studies from our research group. Furthermore, we showed that late lesion growth is associated with worse functional outcome and that IVT does not influence this association between late lesion growth and worse functional outcome.

There are no previous studies assessing the influence of IVT on late lesion growth after treatment. There is little consensus on the influence of IVT on lesion growth in the pre- and post-treatment time window. In two previous studies, lesion growth in the pre- and 24-hour post-treatment window was also comparable between patients that did and did not receive IVT.^9,10^ This is similar to our findings, despite the difference in imaging modality between our study (CT) and theirs (MRI). However, Simonsen et al. showed that the use of IVT reduced infarct growth in a similar time-window. The 24-hour post treatment lesions in their study were evaluated on MRI (63%) and CT (37%) which could have caused heterogeneity in the ischemic constituents considered to be lesion.^11^ Furthermore, Deng et al. use a threshold of 12 ml to define presence of lesion growth, which is closer to the 24-hour lesion volumes we observed, unlike the 50 ml used by Simonsen et al.^9,11^ Nevertheless, it is important to note that patients in these studies did not receive IVT due to contraindications or arriving outside the therapeutic window, unlike our study where patients were randomized to IVT or no IVT.

In our study, we did not find the significant differences in lesion volume and late lesion growth in patients that did and did not receive tPA. Alteplase is a fibrin specific activator that helps in the conversion of plasminogen to plasmin, thereby softening the occluding thrombus. This may facilitate easier thrombectomy or even lysing the residual and/or distal thrombus fragments that can be formed during the intervention, thereby reducing late lesion growth. On the other hand, tPA may also be neurotoxic, especially when the blood flow has not been restored to the downstream territory. In such scenarios, the pressure inside the vessels increases, opening tight junctions within the endothelium allowing the leakage of tPA across the blood-brain barrier and injuring both the neurovascular unit and the brain parenchyma. This may lead to increase in excitotoxicity, more blood-brain barrier injury, and edema.^6,12^

Similar to the findings in our population, several studies have shown that patients with successful treatment have lower lesion volumes at 24 hours and 1 week.^1,3,9–11,13–16^ Unlike a previous study from our group, we did not find any significant differences between late lesion growth between patients with and without successful treatment. This could be due to small number of patients that do not achieve successful treatment (13%) and presence of outliers. Nonetheless, both ischemia and post-ischemic reperfusion can cause disruption of the blood brain barrier, adding to the progression of edema and swelling. This causes a mechanical pressure to the neighboring tissues, leading to a cascade of more edema and ischemia.^1,17^ Furthermore, in the patients that do not receive tPA, due to the absence of the lysing effect of tPA, especially in fibrin-rich clots, more retrieval attempts may be required to successfully recanalize the vessel. As identified by Hassen et al., this may lead to the formation of new emboli, and increase the propagation of ischemia. ^18^ Moreover, the incidence of no-reflow phenomenon is established in animal models, its incidence in humans in controversial. On one hand, Schiphorst et al. found no-reflow phenomenon, defined using ASL imaging and infarction on follow-up MRI, to occur only in one of the 33 patients of their study.^19^ On the other hand, Felix et al. recently performed a pooled analysis of data from the EXTEND-IA, EXTEND-IA TNK and EXTEND-IA TNK part 2 trials and showed that “no-reflow” phenomenon, defined using MR or CT perfusion, occurred in 25% of their cohort (130 patients) and accounted for 60% of the infarct volume.^20^ The influence of thrombus fragmentation, formation of emboli in new territory and reperfusion injury, caused either by hyper-perfusion or no-reflow phenomenon, especially in patients with successful treatment on late lesion volumes and growth could not be assessed in this study due to the small number of patients in the treatment arms and lack of follow-up perfusion imaging.^18,21^

In this study we showed that despite accounting for treatment success and the 24-hour lesion volume, the late, post-treatment lesion growth is still associated with worse clinical outcome. This finding is in line with previous studies from our group.^1,3^ Here, we also showed that receiving IVT does not influence this association. This suggests that the pathophysiological cascade promoting edema and ischemia continues after treatment and is also detrimental to clinical outcome. Our results suggest the need for neuro-protectants and/or secondary therapies that can protect the brain parenchyma from ischemia and post-ischemic reperfusion injuries.^22^ Several targets for neuro-protective management focused at the inflammatory, excitotoxicity and oxidative stress pathways or other non-pharmacological mechanisms are being developed and investigated.^23^ GAMES-RP, a phase II randomized clinical trial showed that intravenous administration of glyburide was associated with reduced edema, midline shift and fewer deaths along with an increase in alertness levels and NIHSS. ^24^ The ESCAPE–NA1 trial, assessing the efficacy of nerinetade, an eicosapeptde, in a multicenter, double-blind, randomized clinical trial showed no added benefit of administering nerinetade on functional outcome. However, accounting to Hill et al., this could be due to the drug-drug interactions of tPA and nerinetade. Accounting for drug-drug interactions, especially in the presence of tPA, is crucial, to prove the beneficial effect of neuro-protectants.^25^

Our study has some limitations. By only including the patients that received NCCT at 24-hours and 1-week, we included less than a third of the trial population. This has led to reduction of statistical power, to assess the effect of thrombus fragmentation and no-reflow phenomenon (especially in the direct IAT treatment arm) on lesion volumes and growth, and validating our group’s previous findings that post-treatment lesion growth is independently associated with an unfavorable functional outcome (mRS ≤ 2).^1,3^ Furthermore, the lack of significant association between baseline NIHSS, ASPECTS and occlusion location with outcome could be due to lack of statistical power, which is further affected by the decreased variation observed in these characteristics due to improved workflow times, especially since MR CLEAN NO-IV trial only enrolled mothership patients. However, we showed that the baseline, clinical and (post-) treatment characteristics of our population are comparable to those excluded from the trial (Supplementary Table 1). Assessing lesions on NCCT is not straightforward, especially in the early time-window of 24 hours when there can be no clear definition of the hypo-dense areas. Moreover, since hemorrhagic areas may appear isodense on NCCT, our delineations that identify hemorrhagic transformation may also be prone to errors. We identified hemorrhagic areas as hyper-dense areas in and around the hypo-dense lesion, thereby using hyper-denisty as a surrogate marker for hemorrhagic transformation. Hence, we cannot claim that our findings regarding hemorrhagic transformation are solely due to bleeding but are due to abnormal hypder-dense areas in and around the lesion. Although this may not be an accurate method to identify and quantify bleeding, hyper-density on NCCT scans still remains the most commonly used modality to quantify hemorrhagic transformation. DWI is a better imaging modality to quantify lesions, in this time-window. But the trial design allowed for a single time-point 24-hour DWI, which restricted studying growth of lesion characteristics over time using this more precise imaging modality. Nonetheless, in our study, the delineations on the 24-hour and 1-week scans were made in accordance with the ASPECTS scores evaluated by the imaging core-lab of the trial and the segmentations were validated with an experienced neuro-radiologist. Interestingly, the baseline, imaging and (post-) treatment characteristics of patients that received the NCCT imaging workflow was comparable to those that received the MR-imaging workflow. Furthermore, distinguishing between lesion growth into the white and grey matter would aid in better understanding the reasons for post-treatment lesion growth. However, this remains outside the scope of this study owing to the difficulty in precisely identifying white and grey matter on NCCTs. The hemorrhage segmentations, especially on the 24-hour scan, include hyper-densities suspected for both hemorrhage and contrast extravasation due to the difficulty of accurately discerning them. Though by assessing only the patients that were identified by the core-lab to suffer from a hemorrhagic transformation on the 1-week scan we mitigate some inaccuracies, this can be a possible explanation for the slightly larger (yet, non-significant) 24-hour hemorrhage volumes.

## Conclusion

We confirmed that late lesion growth in the subacute period (between 24-hours and 1 week) after treatment is common and is associated with poor outcome in the sub-acute period (between 24-hours and 1 week) after treatment. We did not find evidence that IVT with alteplase influences the growth of lesion characteristics. Lastly, IVT did not influence the association between late lesion growth and outcome, suggesting that developing treatments to cease the lesion development are crucial.

## Supplemental Material

sj-pdf-1-jcb-10.1177_0271678X231167755 - Supplemental material for Role of intravenous alteplase on late lesion growth and clinical outcome after stroke treatment Click here for additional data file. Supplemental material, sj-pdf-1-jcb-10.1177_0271678X231167755 for Role of intravenous alteplase on late lesion growth and clinical outcome after stroke treatment by Praneeta Konduri, Fabiano Cavalcante, Henk van Voorst, Leon Rinkel, Manon Kappelhof, Katinka van Kranendonk, Kilian Treurniet, Bart Emmer, Jonathan Coutinho, Lennard Wolff, Jeanette Hofmeijer, Maarten Uyttenboogaart, Wim van Zwam, Yvo Roos, Charles Majoie and Henk Marquering in Journal of Cerebral Blood Flow & Metabolism
