# Supplementary material for: Role of intravenous alteplase on late lesion growth and clinical outcome after stroke treatment
Source: J Cereb Blood Flow Metab. 2023 Apr 5;43(2 Suppl):116–25. doi: 10.1177/0271678X231167755 (PMC10638991; doi:10.1177/0271678X231167755)
Supplement: sj-pdf-1-jcb-10.1177_0271678X231167755 - Supplemental material for Role of intravenous alteplase on late lesion growth and clinical outcome after stroke treatment [file sj-pdf-1-jcb-10.1177_0271678X231167755.pdf]

Supplementary Material

Supplementary Figures

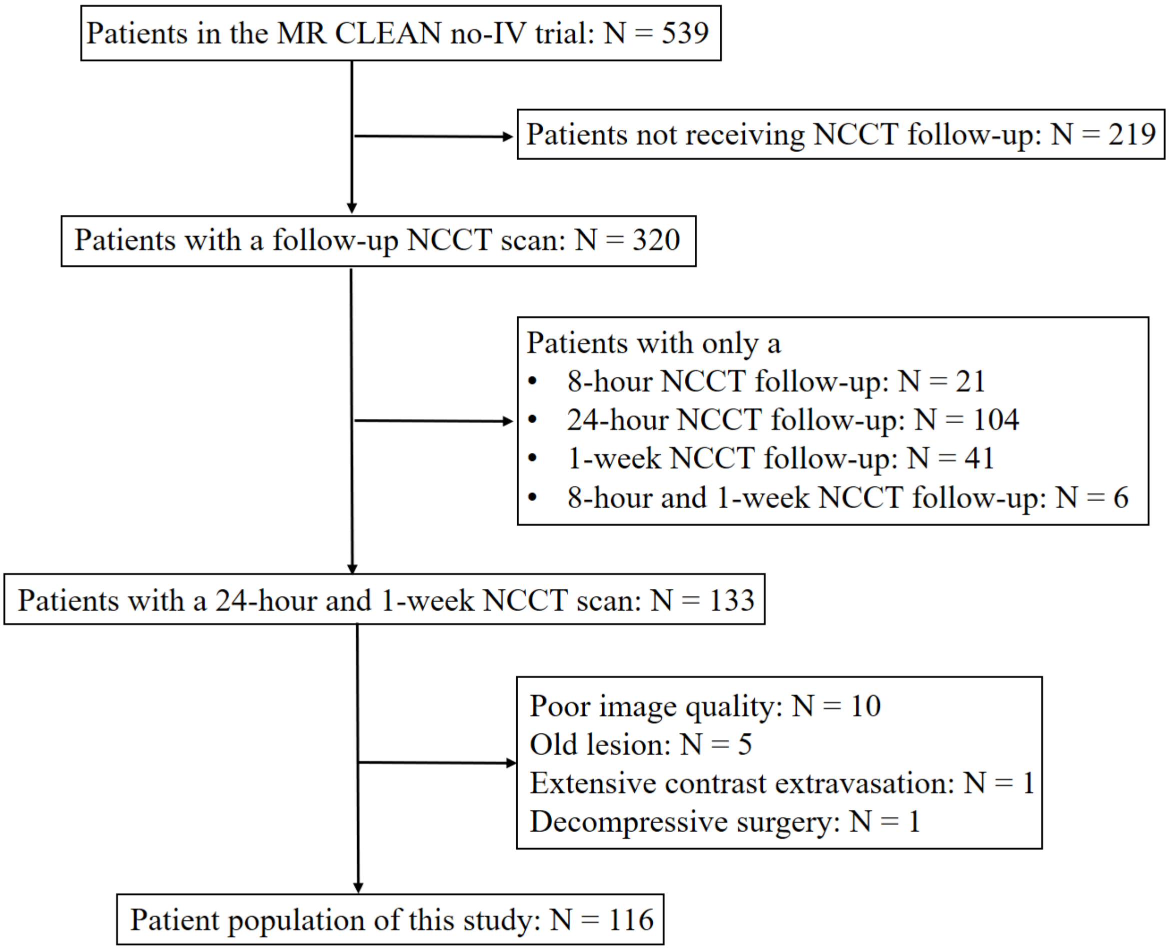

Supplementary Figure 1: Flowchart describing the inclusion and exclusion criterion used in this study

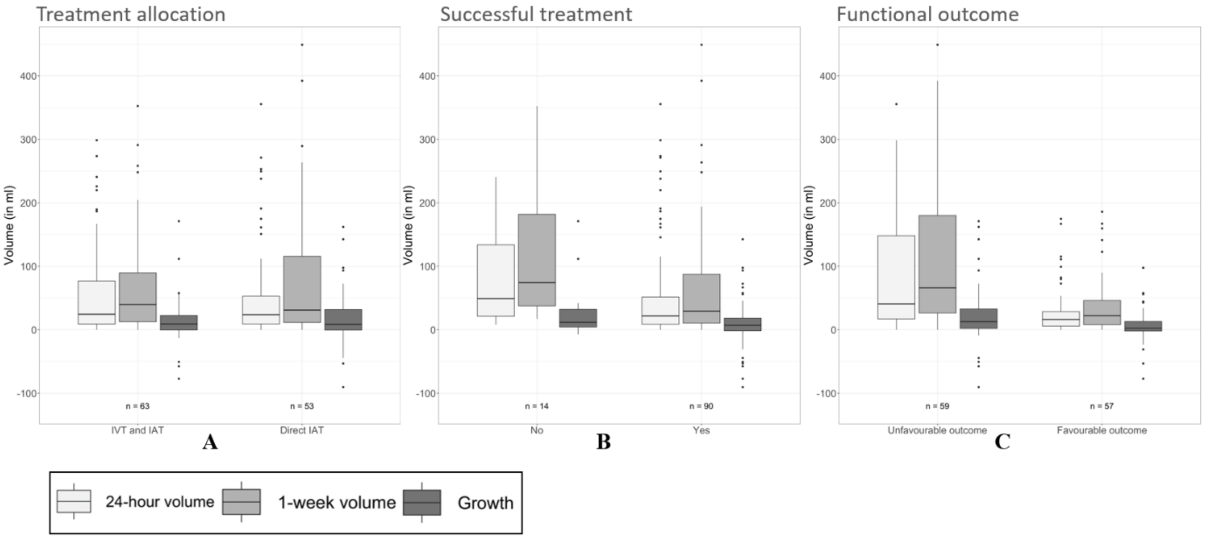

Supplementary Figure 2: Boxplots comparing the 24-hour and 1-week non-hemorrhagic volumes and late non-hemorrhagic growth between subgroups based on (A) treatment allocation, (B) successful treatment (eTICI≥2b) and favourable functional outcome (mrs≤2)

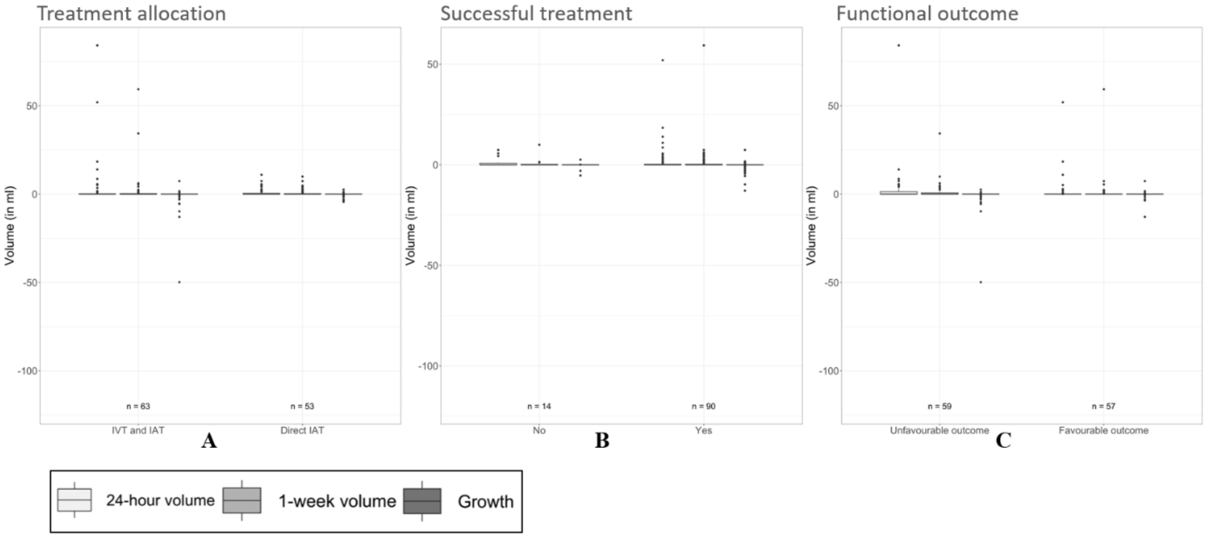

Supplementary Figure 3: Boxplots comparing the 24-hour and 1-week hemorrhagic volumes and late hemorrhagic growth between subgroups based on (A) treatment allocation, (B) successful treatment (eTICI≥2b) and favourable functional outcome (mrs≤2)

Supplementary Tables

Supplementary Table 1: Comparison of baseline, clinical, imaging and (post-) treatment characteristics of MR CLEAN-NO IV trial patients included and excluded in this study

**Characteristics**	**Population (n = 539)**	**Included in study**		**p-value**
		**No (n = 423)**	**Yes (n =116)**	
Age	71(62-79)	71(62-80)	71(59-76)	0.2
Male sex	305(57%)	232(55%)	73(63%)	0.15
**Clinical and imaging characteristics**				

Previous ischemic stroke<br>Atrial fibrillation<br>Diabetes mellitus		91(17%)<br>58(11%)<br>90(17%)	78(18%)<br>40(10%)<br>69(16%)	13(11%)<br>18(16%)<br>21(18%)	0.09<br>0.09<br>0.75
Hypertension	Summary<br>Missing	260(48%)<br>1 (0.19%)	210(50%)	50(43%)	0.24
Pre-treatment mRS >2	Summary<br>Missing	15(2.8%)<br>1(0.19%)	12(2.8%)	3(2.6%)	1
Glucose	Summary<br>Missing	6.7(5.9-7.9)<br>7(1.3%)	6.7(5.9-7.8)	6.6(5.9-8.1)	0.48
Systolic blood pressure	Summary<br>Missing	150(130-170)<br>2(0.37%)	150(130-170)	150(130-170)	0.48
Baseline NIH Stroke Scale<br>ASPECTS		16(10-20)<br>9(8-10)	16(10-20)<br>9(8-10)	16(11-19)<br>9(8-10)	0.54<br>0.07
Collateral score	Absent<br><50%<br>50-100%<br>100%<br>Missing	32(6.1%)<br>152(29%)<br>223(42%)<br>119(23%)<br>13(2.4%)	26(6.3%)<br>121(29%)<br>172(42%)<br>93(23%)	6(5.3%)<br>31(27%)<br>51(45%)<br>26(23%)	0.92
Right hemisphere stroke		249(46%)	192(45%)	57(49%)	0.54
Distal occlusion	Summary<br>Missing	420(78%)<br>1(0.19%)	324(77%)	96(83%)	0.47
**Treatment and post-treatment characteristics**					
Treatment allocation<br>Onset to randomization		273(51%)<br>93(71-140)	220(52%)<br>94(71-150)	53(46%)<br>92(70-140)	0.27<br>0.86
Onset to needle (minutes)	Summary<br>Missing	100(75-160)<br>279(52%)	100(75-160)	92(78-140)	0.89
Door to groin (minutes)	Summary<br>Missing	64(51-78)<br>28(5.2%)	62(50-75)	68(53-90)	0.01
Door to needle (minutes)	Summary<br>Missing	31(24-44)<br>279(52%)	31(23-45)	32(24-40)	0.7
Onset to groin (minutes)	Summary<br>Missing	130(100-180)<br>28(5.2%)	130(100-180)	140(110-190)	0.03
Onset to reperfusion(mins)	Summary<br>Missing	170(140-230)<br>157(29%)	170(140-220)	180(150-230)<br>29(25%)	0.3
Successful recanalization	Summary<br>Missing	388(81%)<br>59(11%)	298(79%)	90(87%)	0.13
Favourable functional outcome		270(50%)	213(50%)	57(49%)	0.9
modified Rankin Scale	0<br>1<br>2<br>3<br>4<br>5<br>6	28(5.2%)<br>57(11%)<br>185(34%)<br>52(9.6%)<br>64(12%)<br>55(10%)<br>98(1%)	25(5.9%)<br>47(11%)<br>141(33%)<br>41(9.7%)<br>46(11%)<br>40(9.5%)<br>83(20%)	3(26%);<br>10(9%);<br>44(38%);<br>11(9.5%)<br>18(16%)<br>15(13%)<br>15(13%)	0.24

Data are displayed as median (interquartile range) or number (%population). Missing information, when applicable, is also provided as number (%population). Mann-Whitney U test and Chi-Square/Fisher tests were performed to compare continuous and binary/categorical variables between the patients included vs. excluded in this study.

Supplementary Table 2: Comparison of baseline, clinical, imaging and (post-) treatment characteristics of patients included in this study and those that received MRI-workflow at follow-up

**Characteristics**		**MRI workflow (n = 205)**	**Included in the study (n = 116)**	**p-value**
Age<br>Male sex		71(62-78)<br>110(54%)	71(59-76)<br>73(63%)	0.35<br>0.13
Previous ischemic stroke<br>Atrial fibrillation<br>Diabetes mellitus<br>Hypertension		33(16%)<br>15(7.3%)<br>24(12%)<br>101(49%)	13(11%)<br>18(16%)<br>21(18%)<br>50(43%)	0.30<br>0.03*<br>0.15<br>0.34
Pre-treatment mRS >2	Summary<br>Missing: 1(0.3%)	1(0.5%)	3(2.6%)	0.27
Glucose	Summary<br>Missing: 3(0.9%)	6.4(5.6-7.6)	6.6(5.9-8.1)	0.13
Systolic blood pressure	Summary	150(130-160)	150(130-170)	0.36
Baseline NIH Stroke Scale<br>ASPECTS		15(9-20)<br>9(8-10)	16(11-19)<br>9(8-10)	0.96<br>0.03
Collateral score	Absent<br><50%<br>50-100%<br>100%<br>Missing: 6(1.9%)	13(6.5%)<br>52(26%)<br>84(42%)<br>52(26%)	6(5.3%)<br>31(27%)<br>51(45%)<br>26(23%)	0.89
Right hemisphere stroke		91(44%)	57(49%)	0.48
Distal occlusion	Summary<br>Missing	157(77%)	96(83%)	0.41
Treatment allocation<br>Onset to randomization		109(53%)<br>93(65-140)	53(46%)<br>92(70-140)	0.24<br>0.64
Onset to needle (minutes)	Summary<br>Missing: 165(51%)	99(69-160)	92(78-140)	0.77
Door to groin (minutes)	Summary<br>Missing: 7(2.2%)	60(49-73)	68(53-90)	<0.01
Door to needle (minutes)	Summary<br>Missing: 165(51%)	29(20-46)	32(24-40)	0.74
Onset to groin (minutes)	Summary<br>Missing: 7(2.2%)	130(100-180)	140(110-190)	0.01
Onset to reperfusion(mins)	Summary<br>Missing: 72(22%)	180(140-230)	180(150-230)	0.49
Successful recanalization	Summary<br>Missing: 24(7.5%)	162(84%)	90(87%)	0.67
Favourable functional outcome		115(56%)	57(49%)	0.28
modified Rankin Scale	0<br>1<br>2<br>3<br>4<br>5<br>6	12(5.8%)<br>20(9.8%)<br>83(40%)<br>26(13%)<br>25(12%)<br>17(8.3%)<br>22(11%)	3(26%);<br>10(9%);<br>44(38%);<br>11(9.5%)<br>18(16%)<br>15(13%)<br>15(13%)	0.53

Data are displayed as median (interquartile range) or number (%population). Missing information, when applicable, is also provided as number (%population). Mann-Whitney U test and Chi-Square/Fisher tests were performed to compare continuous and binary/categorical variables between the patients included vs those that received the follow-up MRI workflow

Supplementary Table 3: Comparison of lesion characteristics between patients that did and did not receive a successful treatment (eTICI:2b-3) in the complete population and in both the treatment arms

	**24 hours**			**1 week**			**Progression**		
**Successful treatment**	**No**	**Yes**	**p-value**	**No**	**Yes**	**p-value**	**No**	**Yes**	**p-value**
Complete population (N = 116)									
**Lesion volume (ml)**	52 (21-140)	22 (8.8-53)	0.05*	75 (38-190)	30 (11-88)	0.02*	12 (4.4-34)	7.2 (-2.1-18)	0.16
**Non-hemorrhagic volume (ml)**	49 (21-130)	22 (8.8-52)	0.04*	75 (38-180)	30 (11-88)	0.02*	12 (4.4-32)	7.2 (-1.6-19)	0.18
**Hemorrhage volume (ml)**	0 (0-0.84)	0 (0-0.29)	0.84	0 (0-0.25)	0 (0-0.3)	0.8	0 (0-0)	0 (0-0)	0.69
Treatment allocation: IVT and EVT (N = 63)									
**Lesion volume (ml)**	69 (18-190)	24 (9.2-48)	0.11	75 (37-200)	31 (10-77)	0.04*	6.2 (4.2-32)	7.1 (-1.9-18)	0.33
**Non-hemorrhagic volume (ml)**	69 (18-190)	24 (9-48)	0.10	75 (37-200)	31 (10-74)	0.03*	6.2 (4.2-32)	7 (-0.63-18)	0.35
**Hemorrhage volume (ml)**	0 (0-0)	0 (0-0.24)	0.71	0 (0-0)	0 (0-0.38)	0.44	0 (0-0.0)	0 (0-0)	0.80
Treatment allocation: Direct EVT (N = 53)									
**Lesion volume (ml)**	46 (41-57)	20 (8.8-57)	0.31	65 (40-96)	27 (11-110)	0.24	19 (8.7-34)	7.4 (-2.4-28)	0.31
**Non-hemorrhagic volume (ml)**	46 (41-53)	20 (8.8-55)	0.28	65 (40-95)	27 (11-110)	0.25	19 (8.7-32)	7.4 (-2.4-28)	0.34
**Hemorrhage volume (ml)**	0 (0-4.4)	0 (0-0.23)	0.37	0 (0-1.4)	0 (0-0.26)	0.56	0 (0-0.0)	0 (0-0)	0.76

Data are displayed as median (interquartile range). Mann-Whitney U test was performed to compare the continuous between the patients. ** p≤0.01, * p≤0.05, ∮ p≤0.10

Supplementary Table 4: Univariable and multivariable ordinal logistic regression models showing the association of late lesion growth between 24 hours and 1 week and modified Rankin Score after 90 days in patients without a hemorrhagic transformation.

Variable	Model 1: Late lesion growth	
	Odds-Ratio (95% CI)	p-value
**Univariable ordinal analysis**		
Late lesion growth	0.76 (0.62-0.90)	<0.01**
**Multivariable ordinal analysis**		
Growth^	0.66(0.51-0.83)	<0.01**
24-hour lesion volume^	0.72(0.61-0.81)	<0.01**
Age	1.00(0.95-1.05)	0.95
Sex (Male)	12.51(3.11-56.10)	<0.01**

Previous ischemic stroke	0.38(0.07-1.91)	0.25
Atrial fibrillation	0.18(0.04-0.80)	0.02*
Systolic blood pressure (mmHg)	1.01(0.99-1.04)	0.26
Onset to randomization (minutes)	0.99(0.98-1.00)	0.12
Successful reperfusion	1.42(0.35-5.83)	0.62

Analysis of late lesion growth is done for 10 ml volume; ** p≤0.01, * p≤0.05

Supplementary Table 5: Univariable ordinal and binary logistic regression of baseline, imaging and (post-) treatment characteristics with modified Rankin Scale (mRS) and favourable functional outcome (mRS≤2) after 90 days for confounder selection

Variable	Ordinal regression		Binary regression	
	Odds ratio (95%CI)	p-value	Odds ratio (95%CI)	p-value
Age	0.95(0.93-0.98)	<0.01**	0.93(0.90-0.97)	<0.01**
Male sex	2.35(1.20-4.66)	0.01*	2.16(1.01-4.75)	0.05*
Clinical characteristics				
Previous ischemic stroke	0.22(0.07-0.67)	0.01*	0.42(0.11-1.38)	0.17
Atrial fibrillation	0.44(0.18-1.07)	0.07∮	0.24(0.07-0.73)	0.02*
Diabetes mellitus	0.82(0.36-1.87)	0.64	0.73(0.28-1.90)	0.53
Hypertension	0.61(0.31-1.19)	0.15	0.60(0.28-1.26)	0.18
Pre-treatment mRS 0-2	0.67(0.06-12.2)	0.75	0.50(0.02-5.36)	0.58
Glucose	1.00(0.88-1.13)	0.99	0.98(0.85-1.14)	0.81
Systolic blood pressure (mmHg)	0.98(0.97-1.00)	0.02*	0.99(0.97-1.00)	0.15
Baseline NIH Stroke Scale	0.99(0.93-1.04)	0.67	0.99(0.93-1.05)	0.73
Imaging characteristics				
ASPECTS	0.95(0.76-1.18)	0.65	0.97(0.76-1.24)	0.82
Collateral score	1.30(0.86-1.96)	0.21	1.13(0.72-1.77)	0.60
Right hemisphere stroke	0.86(0.45-1.66)	0.66	0.87(0.42-1.80)	0.71
Proximal occlusion	2.09(0.86-5.11)	0.10∮	2.02(0.76-5.78)	0.17
Treatment characteristics				
Onset to randomization	0.99(0.99-1.00)	<0.01**	0.99(0.98-1.00)	0.01*
Onset to needle	0.99(0.98-1.00)	0.04*	0.99(0.98-1.00)	0.05*
Door to groin	0.99(0.98-1.00)	0.12	0.99(0.97-1.00)	0.05*
Door to needle	0.98(0.95-1.01)	0.21	0.97(0.9-41.01)	0.15
Onset to groin	0.99(0.99-1.00)	<0.01**	0.99(0.98-1.00)	0.01*
Onset to reperfusion	0.99(0.98-1.00)	<0.01**	0.99(0.98-0.99)	<0.01**
Direct EVT allocation	0.82(0.43-1.59)	0.56	0.75(0.36-1.56)	0.45
Post-treatment characteristics				
Successful treatment	4.57(1.73-12.4)	<0.01**	8.21(2.08-54.8)	0.01*
24-hr lesion volume^	0.89(0.84-0.93)	<0.01**	0.88(0.81-0.94)	<0.01**

^ Analysis done for 10 ml volume; ** p≤0.01, * p≤0.05, ∮ p≤0.10

Supplementary Table 6: Interaction analysis to assess the influence of thrombolytics on late lesion, non-hemorrhagic and hemorrhagic growth on mRS and favourable functional outcome (mRS≤2) after 90 days

Outcome: modified Rankin Scale (mRS) 90 days after stroke onset						
Variable	Late lesion growth		Late non-hemorrhagic growth		Late hemorrhagic growth	
	Odds-Ratio (95% CI)	p-value	Odds-Ratio (95% CI)	p-value	Odds-Ratio (95% CI)	p-value
Growth	0.93(0.82-1.06)	0.25	0.90(0.79-1.03)	0.12	1.11(0.90-1.32)	0.12
Direct EVT	1.08(0.52-2.24)	0.84	1.04(.50-2.16)	0.92	0.77(0.39-1.51)	0.44
Interaction	0.88(0.72-1.06)	0.18	0.90(0.74-1.09)	0.28	1.03(0.59-1.77)	0.93
**Outcome: Favourable functional outcome (mRS≤2) 90 days after stroke onset**						
Variable	Late lesion growth		Late non-hemorrhagic growth		Late hemorrhagic growth	
	Odds-Ratio (95% CI)	p-value	Odds-Ratio (95% CI)	p-value	Odds-Ratio (95% CI)	p-value
Growth	0.92(0.77-1.07)	0.33	0.89(0.73-1.05)	0.21	1.13(0.98-1.49)	0.29
Direct EVT	0.85(0.38-1.89)	0.68	0.81(0.36-1.82)	0.61	0.68(0.32-1.45)	0.32
Interaction	0.94(0.74-1.19)	0.61	0.97(0.75-1.25)	0.80	0.86(0.47-1.56)	0.61

Analysis of late lesion and non-hemorrhagic growth is done for 10 ml volume; ** p≤0.01, * p≤0.05, ∮ p≤0.10

Supplementary Table 7: Multivariable binary logistic regression models showing the association of late lesion and non-hemorrhagic growth with favourable functional outcome (mRS≤2) after 90 days

Variable	Model 1: Late lesion growth		Model 2: Late non-hemorrhagic growth	
	Odds-Ratio (95% CI)	p-value	Odds-Ratio (95% CI)	p-value
Growth^	0.84(0.69-1.00)	0.07∮	0.83(0.68-0.99)	0.06∮
24-hour lesion volume^	0.86(0.77-0.93)	<0.01**	0.86(0.77-0.93)	<0.01**
Age	0.91(0.86-0.96)	<0.01**	0.91(0.86-0.96)	<0.01**
Sex (Male)	1.92(0.64-5.84)	0.24	1.96(0.65-6.00)	0.23
Atrial fibrillation	0.30(0.06-1.33)	0.13	0.30(0.06-1.31)	0.12
Onset to randomization (minutes)	0.99(0.98-1.00)	0.02*	0.99(0.98-1.00)	0.02*
Successful treatment	5.84 (0.98-56.1)	0.08∮	5.90(0.98-57.4)	0.08∮

^ Analysis done for 10 ml volume; ** p≤0.01, * p≤0.05, ∮ p≤0.10

STROBE Statement

	Item No	Recommendation	Page No
**Title and abstract**	1	(*a*) Indicate the study's design with a commonly used term in the title or the abstract	Title page
		(*b*) Provide in the abstract an informative and balanced summary of what was done and what was found	1-2
**Introduction**			
Background/ rationale	2	Explain the scientific background and rationale for the investigation being reported	2-3
Objectives	3	State specific objectives, including any prespecified hypotheses	3
**Methods**			
Study design	4	Present key elements of study design early in the paper	3-4
Setting	5	Describe the setting, locations, and relevant dates, including periods of recruitment, exposure, follow-up, and data collection	3-4
Participants	6	(*a*) Give the eligibility criteria, and the sources and methods of selection of participants. Describe methods of follow-up	3-4, Supplementary Material
		(*b*) For matched studies, give matching criteria and number of exposed and unexposed	NA
Variables	7	Clearly define all outcomes, exposures, predictors, potential confounders, and effect modifiers. Give diagnostic criteria, if applicable	3-5
Data sources/ measurement	8*	For each variable of interest, give sources of data and details of methods of assessment (measurement). Describe comparability of assessment methods if there is more than one group	3-5
Bias	9	Describe any efforts to address potential sources of bias	4-5
Study size	10	Explain how the study size was arrived at	3-4, Supplementary Material
Quantitative variables	11	Explain how quantitative variables were handled in the analyses. If applicable, describe which groupings were chosen and why	4-5
Statistical methods	12	(*a*) Describe all statistical methods, including those used to control for confounding	4-5
		(*b*) Describe any methods used to examine subgroups and interactions	4-5
		(*c*) Explain how missing data were addressed	15-16
		(*d*) If applicable, explain how loss to follow-up was addressed	4-5
		(*e*) Describe any sensitivity analyses	NA
**Results**			
Participants	13*	(a) Report numbers of individuals at each stage of study—eg numbers potentially eligible, examined for eligibility, confirmed eligible, included in the study, completing follow-up, and analysed	3-4, Supplementary Material
		(b) Give reasons for non-participation at each stage	3-4, Supplementary Material
		(c) Consider use of a flow diagram	Supplementary Material

Descriptive data	14*	(a) Give characteristics of study participants (eg demographic, clinical, social) and information on exposures and potential confounders	15-16
		(b) Indicate number of participants with missing data for each variable of interest	15-16
		(c) Summarise follow-up time (eg, average and total amount)	4-5
Outcome data	15*	Report numbers of outcome events or summary measures over time	5-8, Supplementary Material
Main results	16	(*a*) Give unadjusted estimates and, if applicable, confounder-adjusted estimates and their precision (eg, 95% confidence interval). Make clear which confounders were adjusted for and why they were included	5-8, 15-16, Supplementary Material
		(*b*) Report category boundaries when continuous variables were categorized	NA
		(*c*) If relevant, consider translating estimates of relative risk into absolute risk for a meaningful time period	NA
Other analyses	17	Report other analyses done—eg analyses of subgroups and interactions, and sensitivity analyses	6-8
**Discussion**			
Key results	18	Summarise key results with reference to study objectives	8
Limitations	19	Discuss limitations of the study, taking into account sources of potential bias or imprecision. Discuss both direction and magnitude of any potential bias	11-12
Interpretation	20	Give a cautious overall interpretation of results considering objectives, limitations, multiplicity of analyses, results from similar studies, and other relevant evidence	8-12
Generalisability	21	Discuss the generalisability (external validity) of the study results	11
**Other information**			
Funding	22	Give the source of funding and the role of the funders for the present study and, if applicable, for the original study on which the present article is based	12-13

*Give information separately for exposed and unexposed groups.

Note: An Explanation and Elaboration article discusses each checklist item and gives methodological background and published examples of transparent reporting. The STROBE checklist is best used in conjunction with this article (freely available on the Web sites of PLoS Medicine at http://www.plosmedicine.org/, Annals of Internal Medicine at http://www.annals.org/, and Epidemiology at http://www.epidem.com/). Information on the STROBE Initiative is available at http://www.strobe-statement.org.
